# Supplementary material for: Supported Eosin Y as a Photocatalyst for C-H Arylation of Furan in Batch and Flow
Source: Molecules. 2022 Aug 10;27(16):5096. doi: 10.3390/molecules27165096 (PMC9414944; doi:10.3390/molecules27165096)
Supplement: Supplementary file 1 [file molecules-27-05096-s001.zip › molecules-1835869-supplementary.pdf]

Supplementary

Supported Eosin Y as a Photocatalyst for C-H Arylation of Furan in Batch and Flow

Sergio Rossi, Fabian Herbrik, Simonetta Resta and Alessandra Puglisi*

Dipartimento di Chimica, Università degli Studi di Milano, Via Camillo Golgi, 19, 20133 Milano, Italy

Table of Contents

1. General Description of Reagents and Methods pag S2
2. Synthesis and Characterization of Solid-Supported Eosin Y MR-EY pag S4
3. Synthesis of aryl tetrafluoroborates diazonium salts **1a – 1i** pag S7
4. General procedure for the direct C-H arylation of furan with aryl diazonium salts under batch conditions pag S9
5. General procedure for the direct C-H arylation of furan with aryl diazonium salts under flow conditions pag S10
6. NMR spectra pag S13
7. References pag S18

1. General Description of Reagents and Equipment

If not otherwise stated, reagents, solvents and such were used without further purification. Eosin Y was bought from TCI chemicals and was either used without purification or was converted to sodium salt by the addition of aqueous sodium hydroxide solution. *N,N*-dimethylformamide was degassed prior to use. *N,N*-diisopropylethylamine was used without prior purification. Merrifield Resin High Loading 1.2 mmol/g was purchased from Merck. Acetonitrile was used in HPLC grade.

Reactions were monitored by thin-layer chromatography (TLC) on Macherey-Nagel pre-coated silica gel plates (0.25 mm) and visualized by UV light. Flash chromatography was performed on Merck silica gel (60, particle size: 0.040–0.063 mm). ^{1}H NMR, ^{13}C NMR, and ^{19}F NMR spectra were recorded on a Bruker Avance-400 spectrometer in $CDCl_3$ as solvents at room temperature. Chemical shifts for protons are reported using residual solvent protons (^{1}H NMR: δ = 7.26 ppm for $CDCl_3$) as the internal standard. Carbon spectra were referenced to the shift of the ^{13}C signal of $CDCl_3$ (δ = 77.0 ppm). The following abbreviations are used to indicate the multiplicity in NMR spectra: s—singlet; d—doublet; t—triplet; q—quartet; dd—double doublet; ddd—doublet of doublet of doublets; dt—doublet of triplets; m—multiplet; quint—quintuplet; sext—sextuplet; sept—septet; br—broad signal; dq—doublet of quartets.

High-resolution mass spectra (HRMS) were acquired using a Bruker solariX XR Fourier transform ion cyclotron resonance mass spectrometer (Bruker Daltonik GmbH, Bremen, Germany) equipped with a 7 T refrigerated actively shielded superconducting magnet. The samples were ionized in positive ion mode using MALDI or ESI ionization sources.
ATR spectra were recorded using a Jasco FT/IR-4600 instrument.
A KF-Technologies, NE-300 series Just Infusion syringe pump was employed for continuous flow applications.

1.1 Home-made LEDs Photoreactor

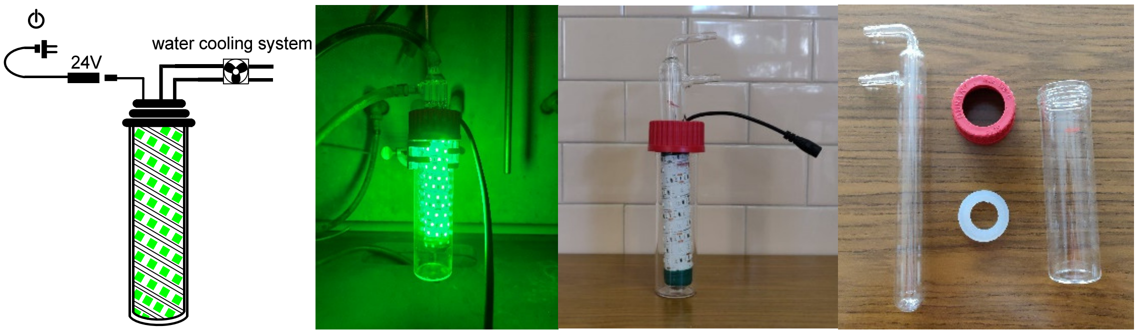

A B C D

Figure S1. (**A**) Water-cooled photoreactor scheme. (**B**) Assembled photoreactor (on). (**C**) Assembled photoreactor (off). (**D**) Unassembled photoreactor.

<u>Construction of the Photoreactor</u>: A making-of video related to the construction of the photoreactor starting from a sublimator apparatus has already been reported in the literature [10]. The central sublimator glass piece is first wrapped to the desired length with heavy-duty aluminum foil to generate a socket for the LED strip that possesses high heat conductive properties. Around this first layer, the LED-strip is then coiled and glued (double-sided adhesive tape), which is further secured in place at the top and bottom with electric isolating tape. The cable is guided through the silicon rubber seal by puncturing it. The final reactor is then assembled as presented in Figure S1.

<u>Green LED characterization:</u> Commercially available SMD LEDs 2835 60 led/m 24 V, with an IP95 "plug and play system" without extra wiring connection, were employed in the realization of the photoreactor shown in Figure 1. The LEDs' wavelength emission profile together with their specific light intensity (expressed as mW/cm^2) were determined using a compact CCD spectrometer (model CCS200/M) connected to a multimode optical fiber, purchased by Thorlabs. As clearly depicted in Figure S2, green LEDs employed are characterized by an almost monochromatic emission profile showing a maximum of intensity located at ca. 512–514 nm. The light power intensity was thus checked using a Thorlabs PM200 power meter equipped with an S130VC power head with an Si detector. The measured light intensities, though slightly decreasing by moving the maximum of LEDs emission towards longer wavelengths, were $I = 423.9 \pm 0.6$ mW/cm^2

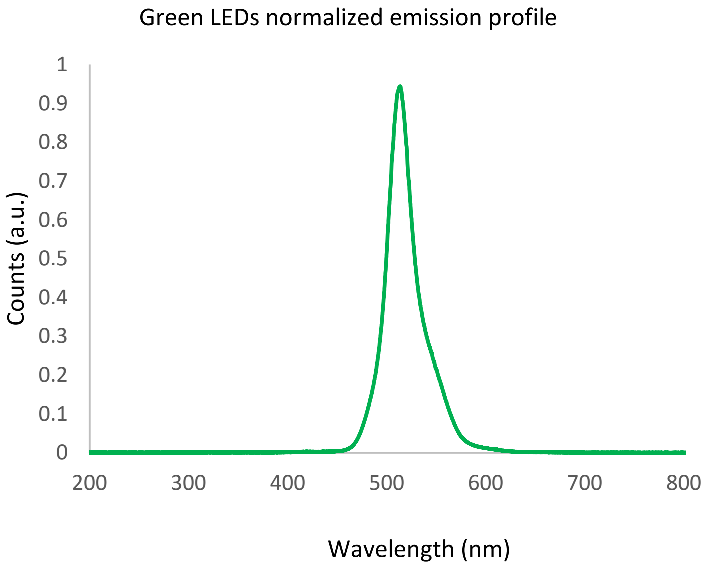

Figure S2. Green LED normalized emission profile.

1.2 Packed-Bed reactor

The packed-bed reactor was realized using a commercially available 100 mm×10 mm (l*ID) Omnifit-Column. The column was filled with 37 glass-balls (OD = 5 mm) and with 1.232 g Eosin Y functionalized Merrifield resin (Figure S3). Regular agitation of the setup ensured complete settling of the solid material inside the reactor. After filling was completed, the closing lid with fluidic connection was reattached. The reactor volume V_R = 1.5 mL was determined by infusing a 0.05 M solution of anthraquinone and periodically (every 50 μL) collecting a drop of the outflow on a piece of TLC plate and checking it using a 254 UV-lamp.

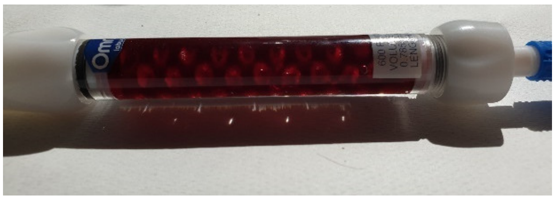

Figure S3. Packed-bed reactor

2. Synthesis and Characterization of Solid-Supported Eosin Y Merrifield Resin (MR-EY)

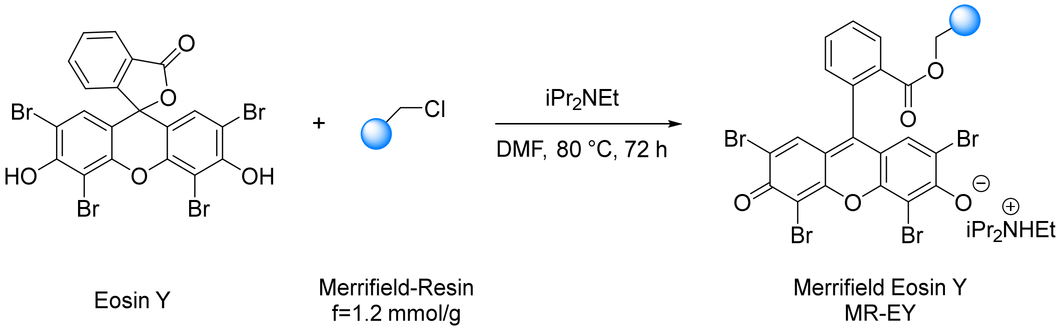

The synthesis was performed according to literature data [11].

In total, 10.0 g (8.33 mmol, 1.00 eq, f=1.20 mmol/g) of Merrifield-Resin High-Load 100-200 mesh was introduced in a 250 mL 3-necked round bottom flask. Then, 6.48 g (10.0 mmol, 1.20 eq) Eosin Y (hydrogen form) was added. The solid was mechanically stirred and 133 mL *N,N*-dimethylformamide was added, followed by 3.48 mL (20.0 mmol, 2.40 eq) diisopropylethylamine. After setting the temperature to 80 °C, the dispersion was stirred for exactly 72 h. After this time, the reaction mixture was poured into an oven-dried glass-sintered funnel (pore size 4, pre-weighed) and special care was taken to remove almost all the material out of the flask with generous amounts of methanol. The residue was infused with a mixture of water/THF/methanol and stirred with a glass rod. After infusing for 5 min, a vacuum was attached, and the washing liquid was filtered off. The vacuum was detached and the whole process was repeated 15 times. After this generous washing process, the process was repeated three times using dichloromethane. The washing flasks was changed and the remains in the funnel were dried by running a constant air stream through them for 5 h by attaching a vacuum. After this time, the filter was weighed again, and by the difference in weight, a preliminary catalyst loading was calculated, which amounted to f = 0.168 mmol/g. The catalyst appeared as a dark red solid.

Elemental analysis of MR-EY: C 80,73, H 6,54, N 0,28.
This corresponds to f = 0.2 mmol/g. The gravimetric loading (f = 0.168 mmol/g) was used in the reactions.

IR

According to Shajan et al. [23], free Eosin-Y presents characteristic vibrational peaks at 1418 (peak #4, Figure S3) and 1744 cm^{-1} (peak #10, Figure S3), which can be assigned to the symmetric vibration of the carboxyl group and carboxyl (C = O) stretching, respectively.

These characteristic vibrational peaks are absent in the functionalized material, meaning that the formation of the ester bond occurs as expected. In addition, two new signals were revealed at 1717 (peak #6, Figure S4) and 1224 cm^{-1} (peak #18, Figure S4), corresponding to the C = O stretching and C-O stretching of an ester group, respectively. IR analysis also revealed the presence of aromatic C-H vibrational stretching at 3023 cm^{-1} (peak #1, Figure S4) and alkyl C-H stretching at 2919 cm^{-1} (peak #2, Figure S4).

The strong vibrational peak at 695 cm^{-1} (peak #31 in Figure S4 and peak #35 in Figure S5) also present in the unfunctionalized Merrifield resin can be ascribed to C-H bending.

FREE EOSIN Y

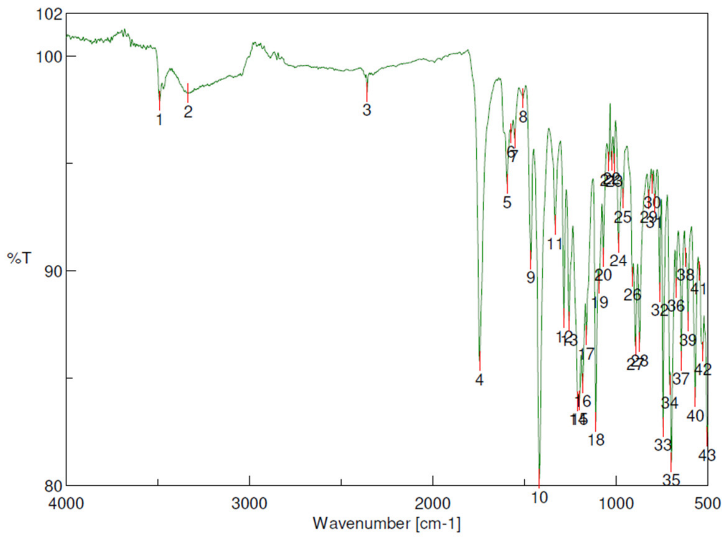

[Result of Peak Picking]

No.	Position	Intensity	No.	Position	Intensity
1	3490.53	97.9091	2	3333.36	98.2743
3	2358.52	98.3286	4	1744.3	85.7776
5	1594.84	94.054	6	1573.63	96.3997
7	1552.42	96.1797	8	1507.1	98.0262
9	1464.67	90.5101	10	1418.39	80.303
11	1332.57	92.1317	12	1284.36	87.7639
13	1256.4	87.6139	14	1209.15	83.9352
15	1200.47	83.9496	16	1183.11	84.7531
17	1161.9	86.9697	18	1110.8	82.9579
19	1091.51	89.376	20	1069.33	90.6198
21	1039.44	95.0855	22	1024.98	95.1521
23	1010.52	95.0133	24	987.375	91.2999
25	962.305	93.3677	26	910.236	89.7067
27	893.844	86.479	28	871.667	86.6542
29	822.491	93.3413	30	802.242	94.0465
31	788.743	93.119	32	761.744	88.9946
33	743.424	82.7185	34	706.783	84.671
35	698.105	81.0845	36	672.071	89.1994
37	644.108	85.7909	38	620.002	90.6393
39	607.467	87.6211	40	567.934	84.1019
41	546.72	90.0026	42	526.471	86.2493
43	503.33	82.2654			

Figure S3. IR of free eosin Y.

MR-EY

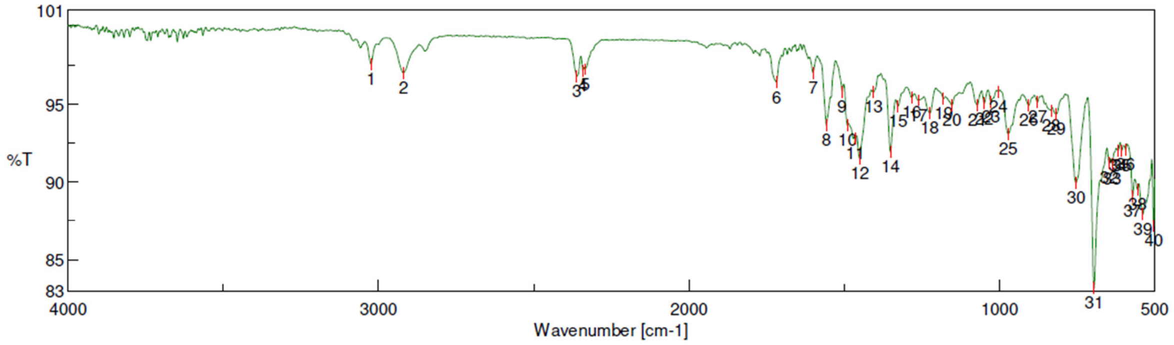

[Result of Peak Picking]

No.	Position	Intensity	No.	Position	Intensity	No.	Position	Intensity
1	3023.84	97.5592	2	2919.7	96.9888	3	2361.41	96.7718
4	2342.12	97.1268	5	2334.41	97.224	6	1717.3	96.3687
7	1600.63	96.9942	8	1557.24	93.6147	9	1507.1	95.7922
10	1489.74	93.622	11	1464.67	92.7665	12	1450.21	91.5185
13	1405.85	95.7442	14	1350.89	91.9488	15	1326.79	94.8462
16	1282.43	95.4103	17	1261.22	95.228	18	1224.58	94.3995
19	1180.22	95.3324	20	1153.22	94.909	21	1071.26	94.9232
22	1049.09	95.027	23	1027.87	95.0948	24	1003.77	95.7739
25	971.947	93.0639	26	905.415	94.898	27	877.452	95.1067
28	834.062	94.5835	29	816.706	94.3724	30	753.066	89.9353
31	695.212	83.1736	32	647.001	91.2193	33	637.358	91.1784
34	618.074	92.0092	35	605.539	92.0167	36	593.968	92.0397
37	570.826	89.0589	38	555.398	89.5108	39	538.042	87.9167
40	503.33	87.1426						

Figure S4. IR of Merrifield resin functionalized with Eosin Y.

MERRIFIELD RESIN

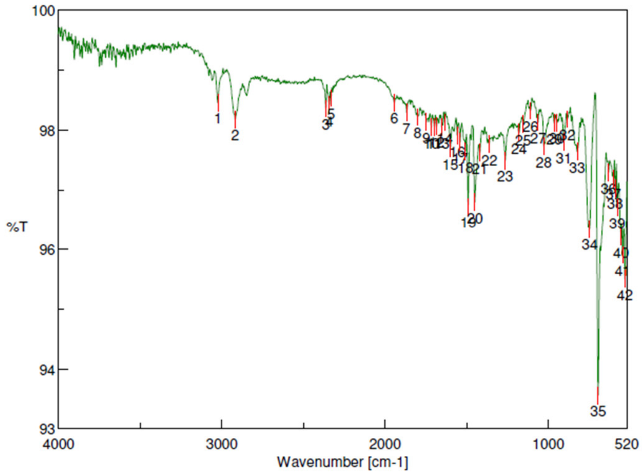

[Result of Peak Picking]

No.	Position	Intensity	No.	Position	Intensity
1	3024.8	98.4576	2	2917.77	98.1782
3	2363.34	98.3592	4	2342.12	98.4145
5	2331.52	98.5331	6	1943.89	98.4564
7	1867.72	98.2918	8	1801.19	98.2166
9	1748.16	98.1347	10	1717.3	98.0474
11	1698.02	98.0488	12	1684.52	98.0611
13	1653.66	98.0505	14	1636.3	98.1273
15	1600.63	97.6927	16	1558.2	97.9059
17	1540.85	97.7991	18	1508.06	97.6418
19	1491.67	96.713	20	1451.17	96.7923
21	1419.35	97.6202	22	1362.46	97.7689
23	1264.11	97.4886	24	1180.22	97.9524
25	1154.19	98.1003	26	1110.8	98.3229
27	1063.55	98.1284	28	1027.87	97.7325
29	964.233	98.1195	30	947.842	98.1235
31	906.379	97.7965	32	884.202	98.1808
33	823.455	97.64	34	747.281	96.3441
35	695.212	93.5571	36	631.573	97.2916
37	600.717	97.1883	38	591.075	97.032
39	578.54	96.7028	40	555.398	96.2056
41	545.756	95.9062	42	531.293	95.5026

Figure S5. IR of unfunctionalized Merrifield resin.

3. Synthesis of aryl tetrafluoroborates diazonium salts 1a – 1i

PROCEDURE A [12]

The desired aniline (10 mmol) was dissolved in a mixture of 4 mL distilled water and 3.35 mL 47% hydrofluoroboric acid. After cooling the reaction mixture to 0 °C using an ice bath, a solution of sodium nitrite (10 mmol, 0.69 g) in 1.5 mL H_2O was added dropwise in 15 min. The resulting mixture was stirred for 1 h and the precipitate was collected by filtration. The crude product was then dissolved in the minimum amount of acetone, and diethyl ether was added until the precipitation of diazonium tetrafluoroborate was completed. The solid obtained was then filtered and washed with diethyl ether (3 × 15 ml) and dried under vacuum.

PROCEDURE B [17]

The desired aniline (10 mmol) was dissolved in 10 mL HCl 3M. The resulting suspension was cooled to 0 °C and then a solution of sodium nitrite (22 mmol, 1,52 g) in 2.5 mL H_2O was added dropwise in 15 min. The reaction was stirred for 15 min at 0 °C and then the crude mixture was washed twice with cold Et_2O (2×15 mL). The aqueous phase was recovered and treated with 6 mL 47% hydrofluoroboric acid. The resulting solution was extracted twice with 15 mL dichloromethane and then the organic phases were combined, washed with 10 mL water, dried with Na_2SO_4, and dried under vacuo to provide the arenediazonium salts as viscous oil. No further purification was required.

4-Chloro-phenyl diazonium tetrafluoroborate 1a

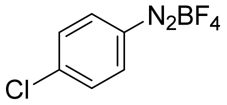

Obtained according to procedure A in a 96% yield (white solid). Data in agreement with those reported in the literature [12].

^{1}H- NMR (300 MHz, d_6-DMSO): 8.70 d (2H, J = 9 Hz), 8.12 d (2H, J = 9 Hz).

4-Methoxy-phenyl diazonium tetrafluoroborate 1b

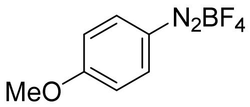

Obtained according to procedure A in a 90% yield (white solid). Data in agreement with those reported in the literature [12].

^{1}H- NMR (300 MHz, d_6-DMSO): 8.61 d (2H, J = 9.4 Hz) 7.48 d (2H, J = 9.4 Hz), 4.04 s (3H).

4-Nitro-phenyl diazonium tetrafluoroborate 1c

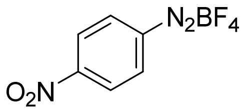

Obtained according to procedure A in a 72% yield (white solid). Data in agreement with those reported in the literature [12].

^{1}H- NMR (300 MHz, d_6-DMSO): 8.93 d (2H, J = 9 Hz), 8.72 d (2H, J = 9 Hz).

4-butyl-phenyl diazonium tetrafluoroborate 1d

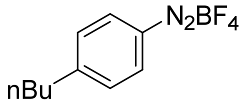

Obtained according to procedure B in an 88% yield (light brown oil).

^{1}H- NMR (300 MHz, $CDCl_3$): 8.51 d (2H, J = 9 Hz), 7.59 (2H, J = 9 Hz), 2.80 t (2H, J = 6 Hz), 1.67-1.57 m (2H), 1.42-1.30 m (2H), 0.92 t (3H, J = 6 Hz).

3,4,5-trimethoxy-phenyl diazonium tetrafluoroborate 1e

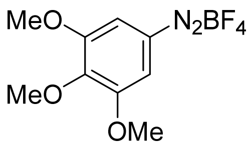

Obtained according to procedure A in a 95% yield (white solid). Data in agreement with those reported in the literature [24].

^{1}H- NMR (300 MHz, d_6-DMSO): 8.19 s (2H), 4.04 s (3H), 3.91 s (6H).

3,5-dichloro-phenyl diazonium tetrafluoroborate 1f

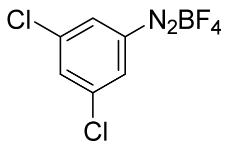

Obtained according to procedure A in a 92% yield (white solid). Data in agreement with those reported in the literature [25].

^{1}H- NMR (300 MHz, d_6-DMSO): 8.83 s (2H), 8.63 s (1H).

2-Methoxy-phenyl diazonium tetrafluoroborate 1g

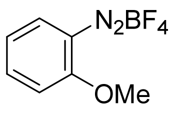

Obtained according to procedure A in a 75% yield (white solid). Data in agreement with those reported in the literature [26].

^{1}H- NMR (300 MHz, d_6-DMSO): 8.52 d (1H, J = 9 Hz), 8.24 t (1H, J = 9 Hz), 7.69 d (1H, J = 9 Hz), 7.45 t (1H, J = 9 Hz), 4.20 s (3H).

3-Nitro-phenyl diazonium tetrafluoroborate 1h

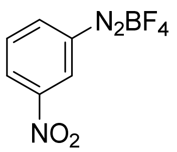

Obtained according to procedure A in an 88% yield (white solid). Data in agreement with those reported in the literature [12].

^{1}H- NMR (300 MHz, d_6-DMSO): 9.63 s (1H), 9.02 t (3H, *J* = 9 Hz), 8.25 t (3H, *J* = 9 Hz).

2-chloro-4-nitro-phenyl diazonium tetrafluoroborate 1i

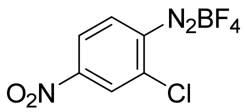

Obtained according to procedure A in a 30% yield (white solid). Data in agreement with those reported in the literature [27].

^{1}H- NMR (300 MHz, d_6-DMSO): 9.04 s (1H), 8.59 dd (1H, *J* = 3 Hz, *J* = 9 Hz), 8.46 d *J* = 9 Hz).

4. General procedure for the direct C-H arylation of furan with aryl diazonium salts under batch conditions

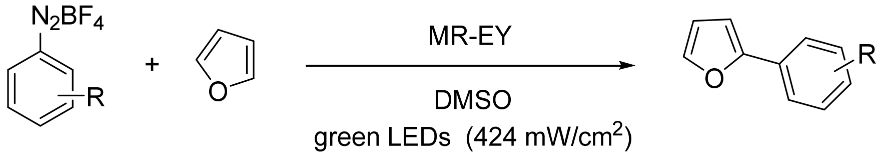

3a-i

A 5 mL vial equipped with a magnetic stirring bar was loaded with MR-Eosin Y (0.02 equiv, 0.013 mmol, 77.3 mg), the desire aryl diazonium tetrafluoroborate (1 equiv, 0.662 mmol), and 2.6 mL dry DMSO. The resulting mixture was degassed by "pump-freeze-thaw" cycles (×2) via a syringe needle and then furan (20 equiv, 13.24 mmol, 970 µL) was added. The vial was attached with a rubber band to the wall of the photoreactor and irradiated using green LEDs for 2–24 h. After the desired time, the reaction mixture was quenched with water (1.5 mL) and filtered with a Millipore apparatus (0.1 um hydrophilic filter). MR-EY resin was recovered, and the crude mixture was then extracted with diethyl ether (5×10 ml) and the combined organic layers were dried over $NaSO_4$, filtered, and concentrated under vacuum.

Purification of the crude product was achieved by flash column chromatography using different mixtures of hexane:ethyl acetate as the eluent.

5. General procedure for the direct C-H arylation of furan with aryl diazonium salts under flow conditions

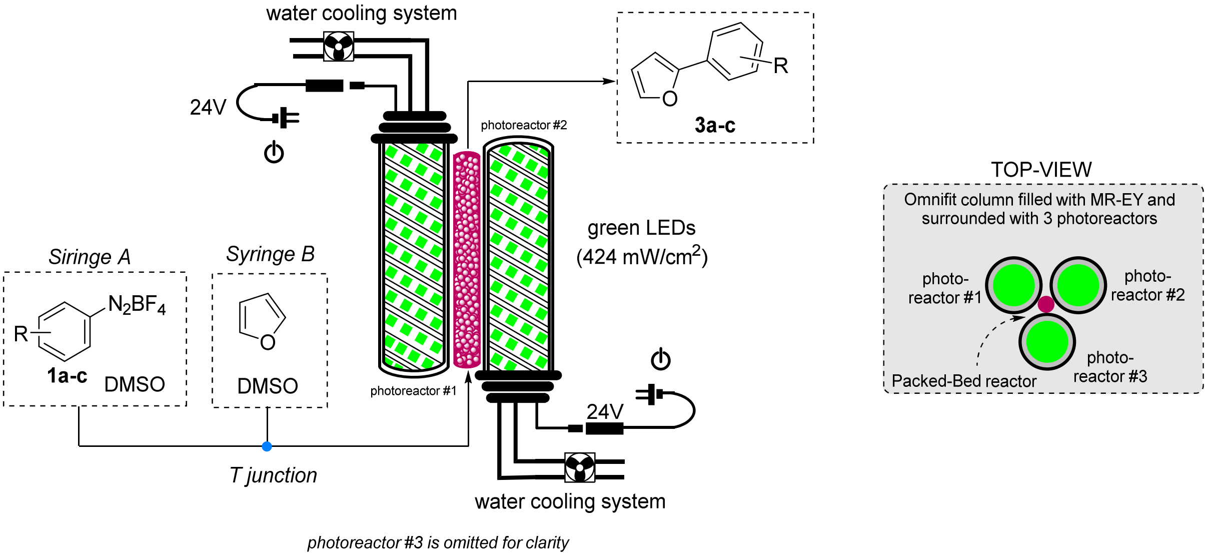

The system consisted of a packed-bed reactor surrounded with 3 equidistant photoreactors. The Omnifit column was positioned vertically and fed from the bottom to the top using a syringe pump.

In a typical experiment, syringe A was filled with a mixture obtained by dissolving 3.6 mmol desire aryl diazonium tetrafluoroborate in 10 ml degassed DMSO (final concentration 0.36 M). Syringe B was loaded with a solution obtained by diluting 5.43 mL furan in 4.57 mL degassed DMSO (final concentration 7.42 M). Mixtures A and B were pumped into the packed-bed reactor at the desired flow rate using a syringe pump. Two reactor volumes were discarded before starting sample collection in order to achieve steady-state conditions. Different samples were collected according to the residence time into a vial containing 1.5 ml water and protected from sunlight with aluminum foil. The crude mixture was then extracted with diethyl ether (5×10 ml) and the combined organic layers were dried over $NaSO_4$, filtered, and concentrated under vacuum. Purification of the crude product was achieved by flash column chromatography using different mixtures of hexane:ethyl acetate as the eluent.

Yield was calculated according to the formula:

$$yield = \frac{mmol_{3a-c}}{flowrate\left(\frac{uL}{min}\right) * concentration_{3a-c}\left(\frac{mmol}{mL}\right) * \frac{1\ mL}{1000\ uL} * collecting_{time}\ (min)} * 100$$

2-(4-Chloro-phenyl)-furan (3a)

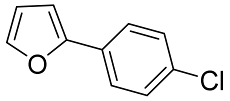

Isolated by chromatographic purification (eluent: hexane) as a white solid. Data in agreement with those reported in the literature [12].

^{1}H- NMR (300 MHz, $CDCl_3$): 7.62 d (2H, *J* = 8.6 Hz), 7.49 d (1H, *J* = 1.6 Hz), 7.37 d (2H, *J* = 8.6 Hz), 6.66 d (1H, *J* = 3.4 Hz), 6.49 dd (1H, *J* = 3.4, 1.8 Hz).

2-(4-methoxy-phenyl)-furan (3b)

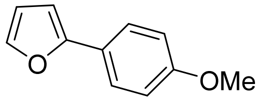

Isolated by chromatographic purification (hexane:AcOEt 98:2) as a white solid. Data in agreement with those reported in the literature [12].

^{1}H- NMR (300 MHz, $CDCl_3$): 7.63 d (2H, J = 8.9 Hz), 7.45 d (1H, *J* = 1.2 Hz), 6.95 d (2H, *J* = 8.9 Hz), 6.54 d (1H, *J* = 3.3 Hz), 6.47 dd (1H, *J* = 3.3, 1.8 Hz), 3.86 s (3H).

2-(4-nitro-phenyl)-furan (3c)

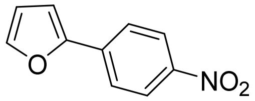

Isolated by chromatographic purification (hexane:AcOEt 95:5) as a yellow solid. Data in agreement with those reported in the literature [12].

^{1}H- NMR (300 MHz, $CDCl_3$): 8.27 d (2H, *J* = 9.0 Hz), 7.82 d (2H, *J* = 9.0 Hz), 7.60 s (1H), 6.90 d (1H, *J* = 3.3 Hz), 6.58 s (1H).

2-(4-butyl-phenyl)-furan (3d)

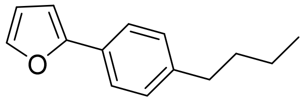

Isolated by chromatographic purification (eluent: hexane:AcOEt 95:5) as a yellow liquid.

^{1}H- NMR (300 MHz, $CDCl_3$): 7.63 d (2H, *J* = 8.2 Hz), 7.48 s (1H), 7.26 d (2H, *J* = 8.2 Hz), 6.63 d (1H, *J* = 3.35 Hz), 6.50 dd (1H, *J* = 3.35, 1.8 Hz), 2.67 t (2H, *J* = 7.5 Hz), 1.69-1.64 m (2H), 1.46-1.18 m (2H), 0.99 t (2H, *J* = 7.5 Hz).

^{13}C- NMR (300 MHz, $CDCl_3$): 154.3, 142.2, 141.7, 128.7, 128.5, 123.8, 111.5, 104.2, 35.44, 33.57, 23.4, 14.0.

2-(3,4,5-trimethoxy-phenyl)-furan (3e)

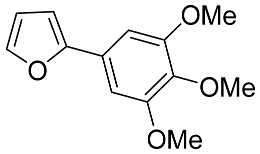

Isolated by chromatographic purification (eluent: hexane:AcOEt 95:5) as a white solid. Data in agreement with those reported in the literature [18].

^{1}H- NMR (300 MHz, $CDCl_3$): 7.47 d (1H, *J* = 0.5 Hz), 6.92 s (2H), 6.59 d (1H, *J* = 3.35 Hz), 6.48 dd (1H, *J* = 3.35, 1.8 Hz), 3.93 s (6H), 3.89 s (3H).

2-(3,5-dichloro-phenyl)-furan (3f)

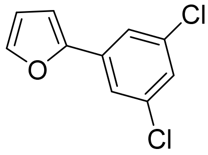

Isolated by chromatographic purification (eluent: hexane) as a white solid. Data in agreement with those reported in the literature [19].

^{1}H- NMR (300 MHz, $CDCl_3$): 7.56 s (2H), 7.25 s (1H), 6.71 d (J = 3.4 Hz) 6.51 dd (J = 3.4, 1.8 Hz).

2-(2-methoxy-phenyl)-furan (3g)

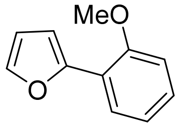

Isolated by chromatographic purification (eluent: hexane:AcOEt 95:5) as a white solid. Data in agreement with those reported in the literature [20].

^{1}H- NMR (300 MHz, $CDCl_3$): 7.90 d (1H, *J* = 7.5 Hz), 7.50 s (1H), 7.25 td (1H, *J* = 7.5, 1.65 Hz), 7.06 t (1H, *J* = 7.5 Hz), 7.01-6.98 m (2H), 6.53 dd (1H, *J* = 3.2, 1.9 Hz), 3.97 s (1H).

2-(3-nitro-phenyl)-furan (3h)

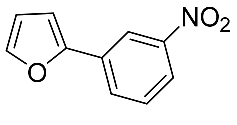

Isolated by chromatographic purification (hexane:AcOEt 95:5) as a yellow solid. Data in agreement with those reported in the literature [12].

^{1}H- NMR (300 MHz, $CDCl_3$): 8.50-8.49 m (1H), 8.09 dd (1H, *J* = 8.2, 2.2 Hz), 7.96 d (1H, *J* = 8.2 Hz), 7.58-7.53 m (2H), 6.82 d, (1H, *J* = 3.4 Hz), 6.54 dd (1H, *J* = 3.4, 1.8 Hz).

2-(2-chloro-4-nitro-phenyl)-furan (3i)

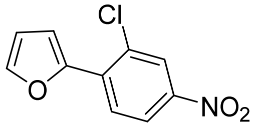

Isolated by chromatographic purification (hexane:AcOEt 95:5) as a white solid. Data in agreement with those reported in the literature [21].

^{1}H- NMR (300 MHz, $CDCl_3$): 8.33 d (1H, *J* = 2.25 Hz), 8.17 dd (1H, *J* = 8.85, 2.25 Hz), 8.07 d (1H, *J* = 8.85 Hz) 7.64-7.63 m (1H), 7.43 d (1H, *J* = 3.57 Hz), 6.63 dd (1H, *J* = 3.57, 1.8 Hz).

NMR spectra

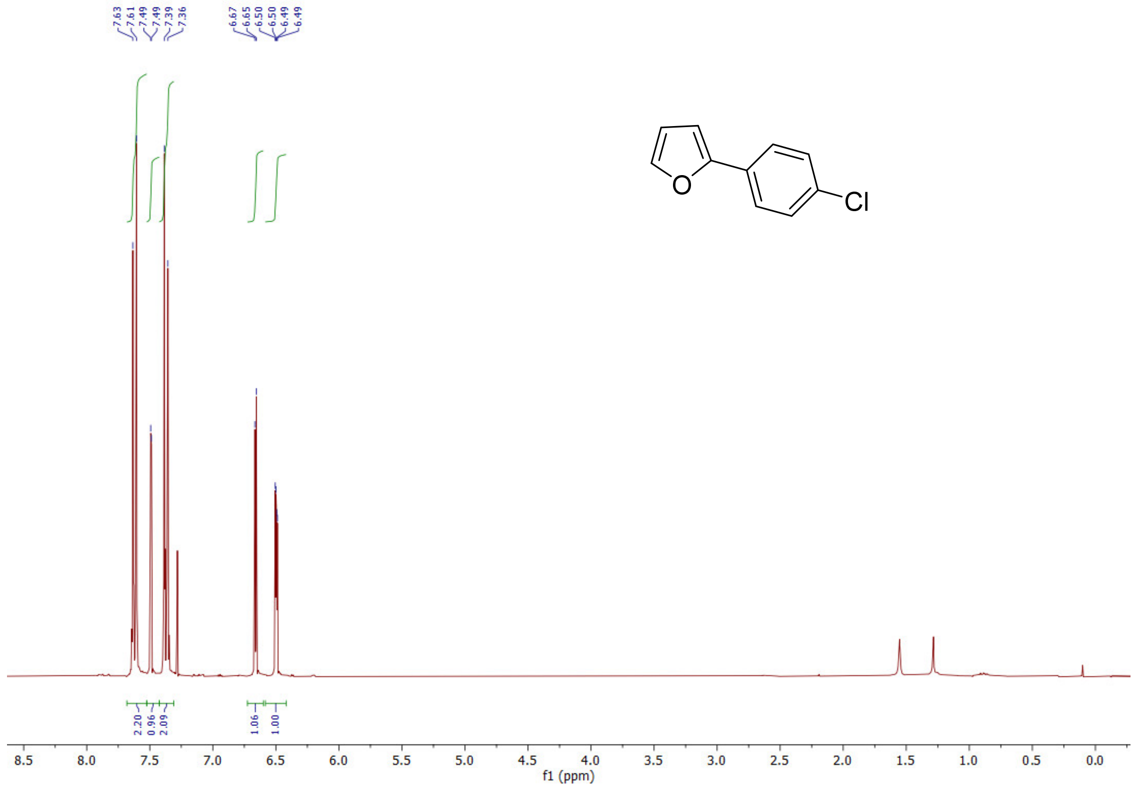

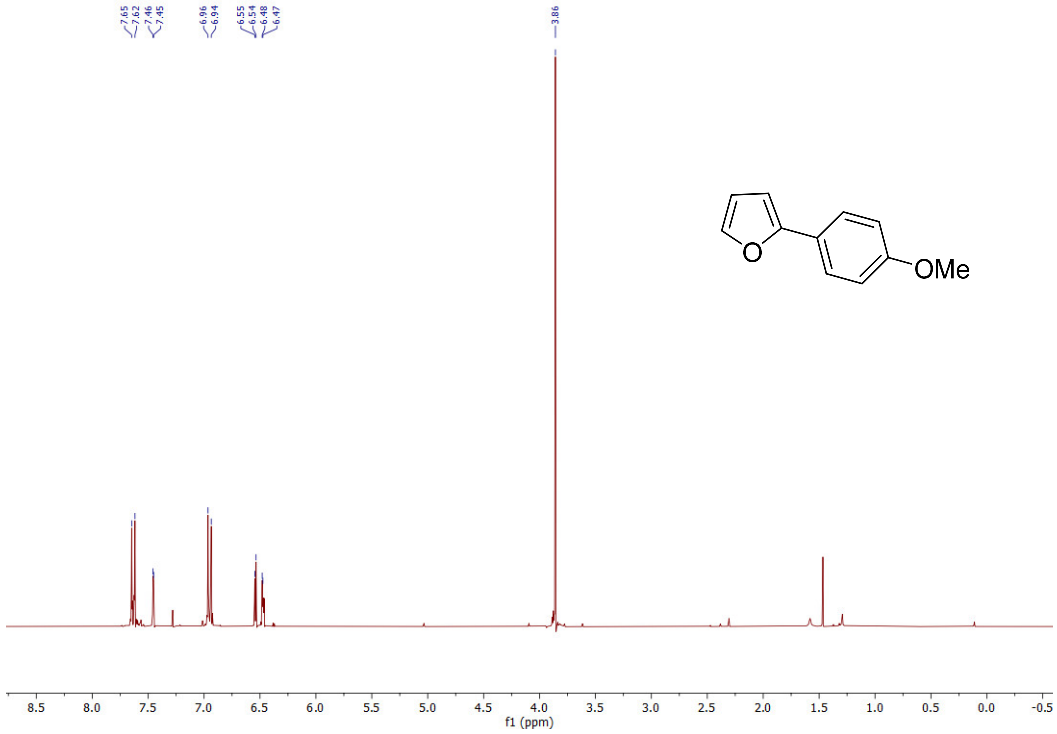

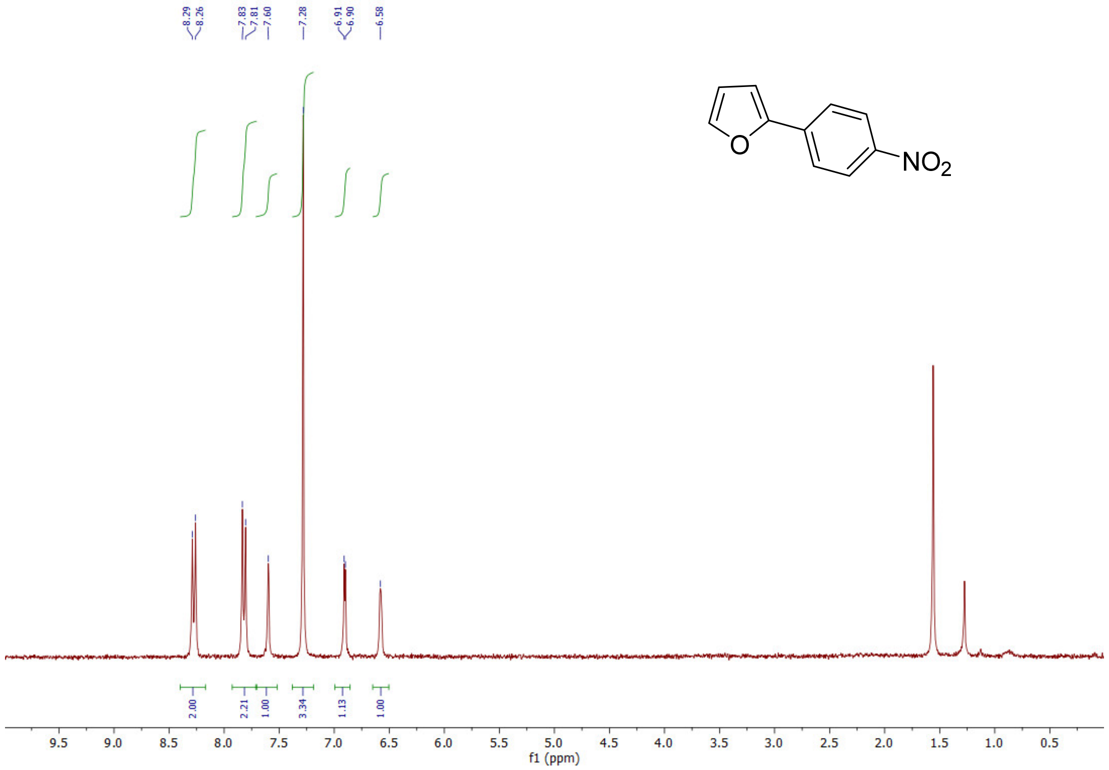
8.29
8.26
7.83
7.81
7.60
7.28
6.91
6.90
6.58
NO2
O
2.00
2.21
1.00
3.34
1.13
1.00
9.5 9.0 8.5 8.0 7.5 7.0 6.5 6.0 5.5 5.0 4.5 4.0 3.5 3.0 2.5 2.0 1.5 1.0 0.5
f1 (ppm)

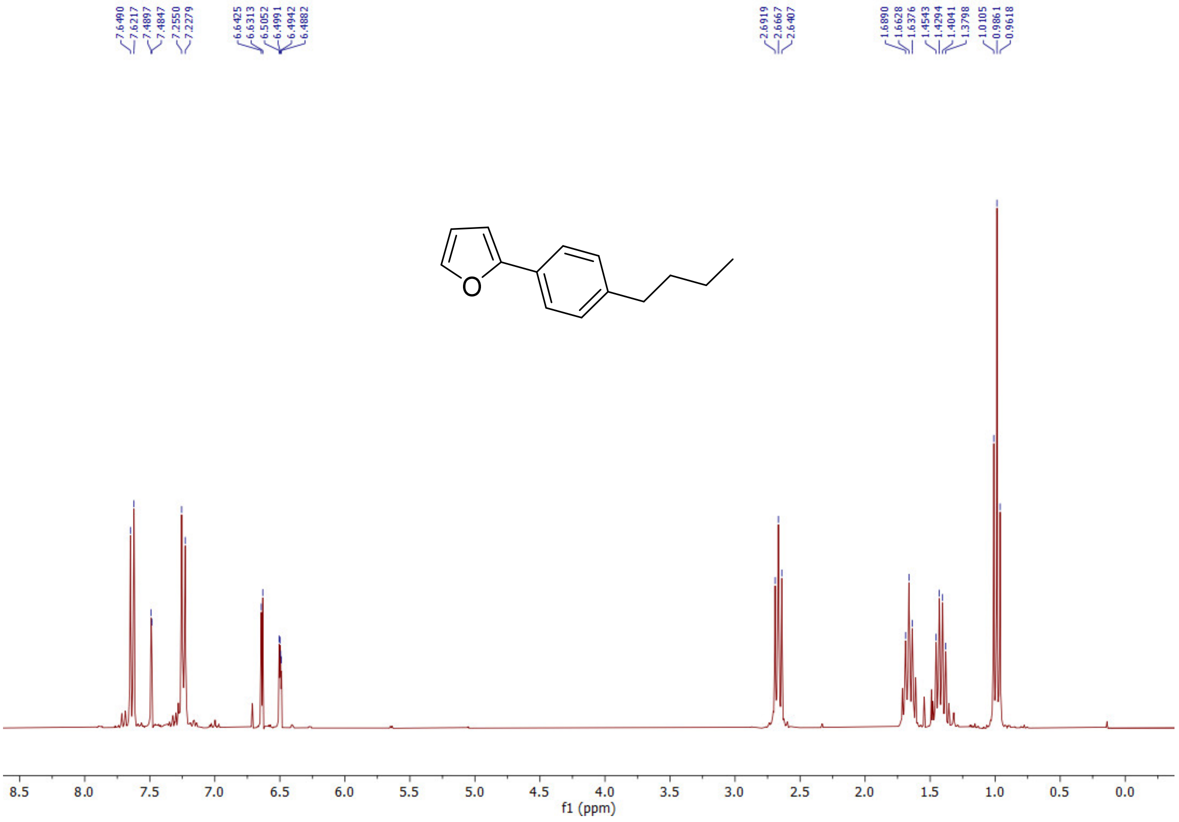
7.6490
7.6217
7.4897
7.4847
7.2550
7.2279
6.6425
6.6313
6.5052
6.4991
6.4942
6.4882
2.6919
2.6667
2.6407
1.6890
1.6628
1.6376
1.4543
1.4294
1.4041
1.3798
1.0105
0.9861
0.9618
O
8.5 8.0 7.5 7.0 6.5 6.0 5.5 5.0 4.5 4.0 3.5 3.0 2.5 2.0 1.5 1.0 0.5 0.0
f1 (ppm)

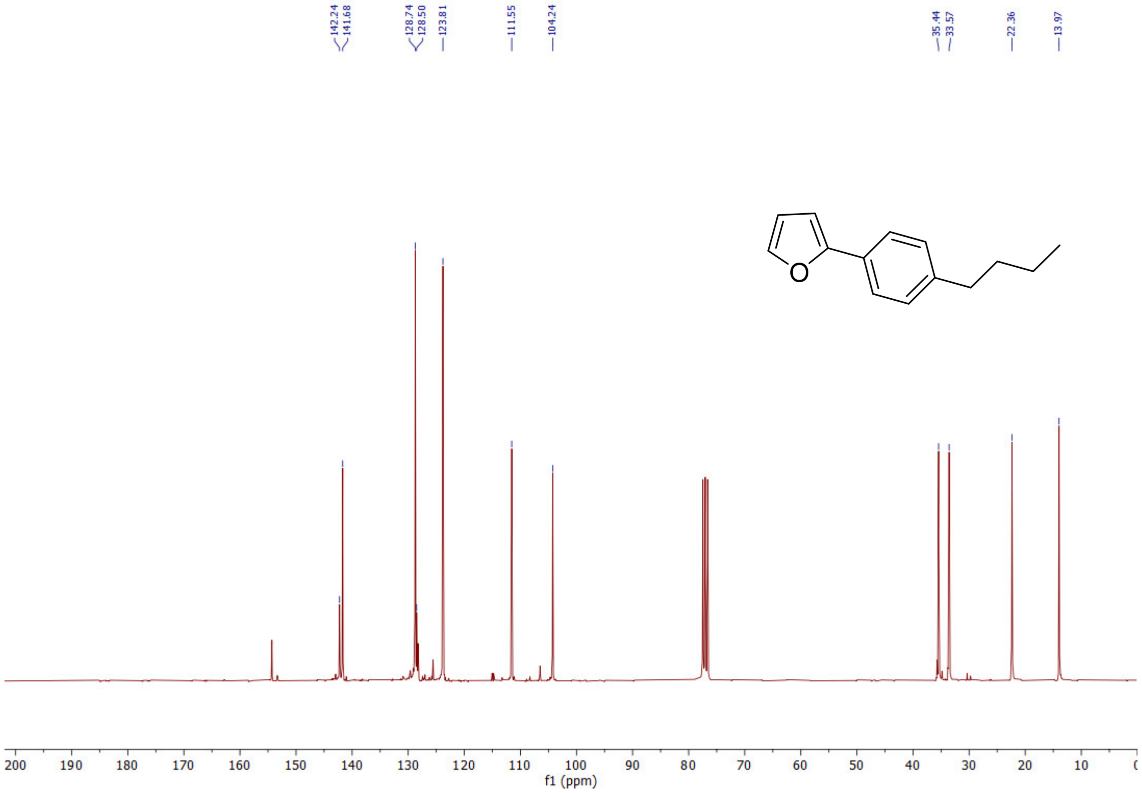
142.24
141.68
128.74
128.50
123.81
111.55
104.24
35.44
33.57
22.36
13.97
O
200 190 180 170 160 150 140 130 120 110 100 90 80 70 60 50 40 30 20 10
f1 (ppm)

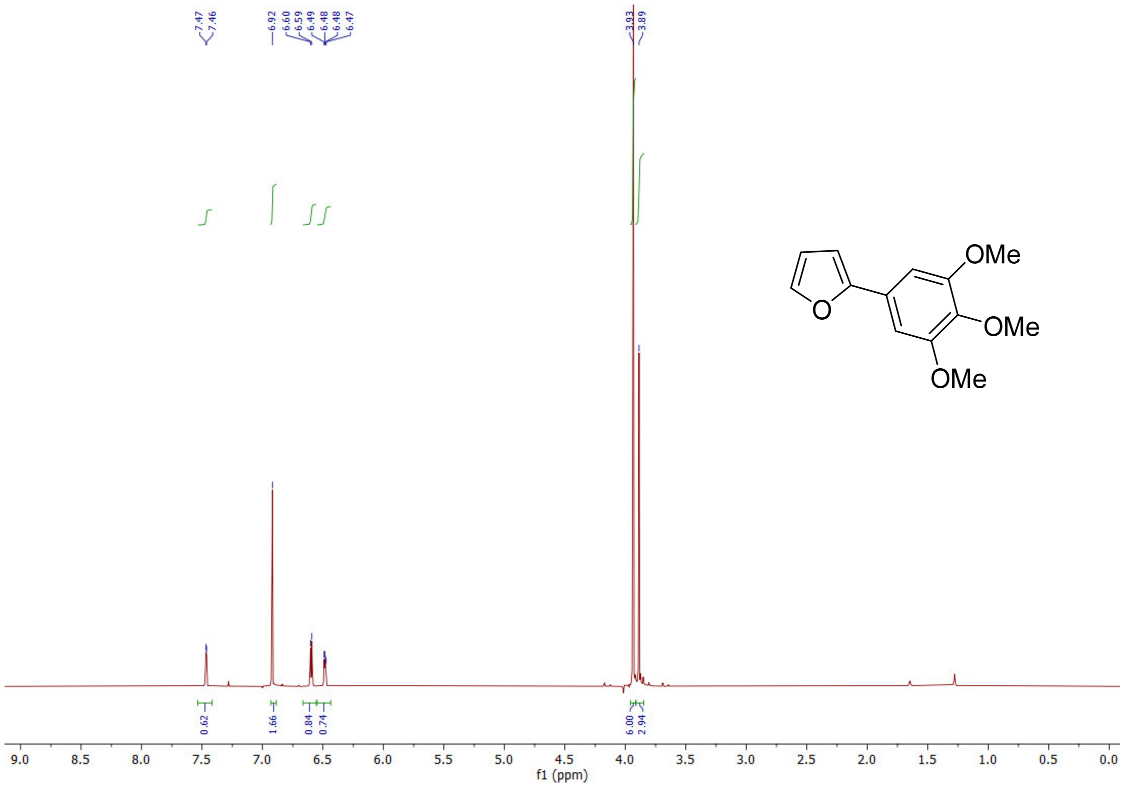
7.47
7.46
6.92
6.60
6.59
6.49
6.48
6.48
6.47
3.93
3.89
O
OMe
OMe
OMe
0.62
1.66
0.84
0.74
6.00
2.94
9.0 8.5 8.0 7.5 7.0 6.5 6.0 5.5 5.0 4.5 4.0 3.5 3.0 2.5 2.0 1.5 1.0 0.5 0.0
f1 (ppm)

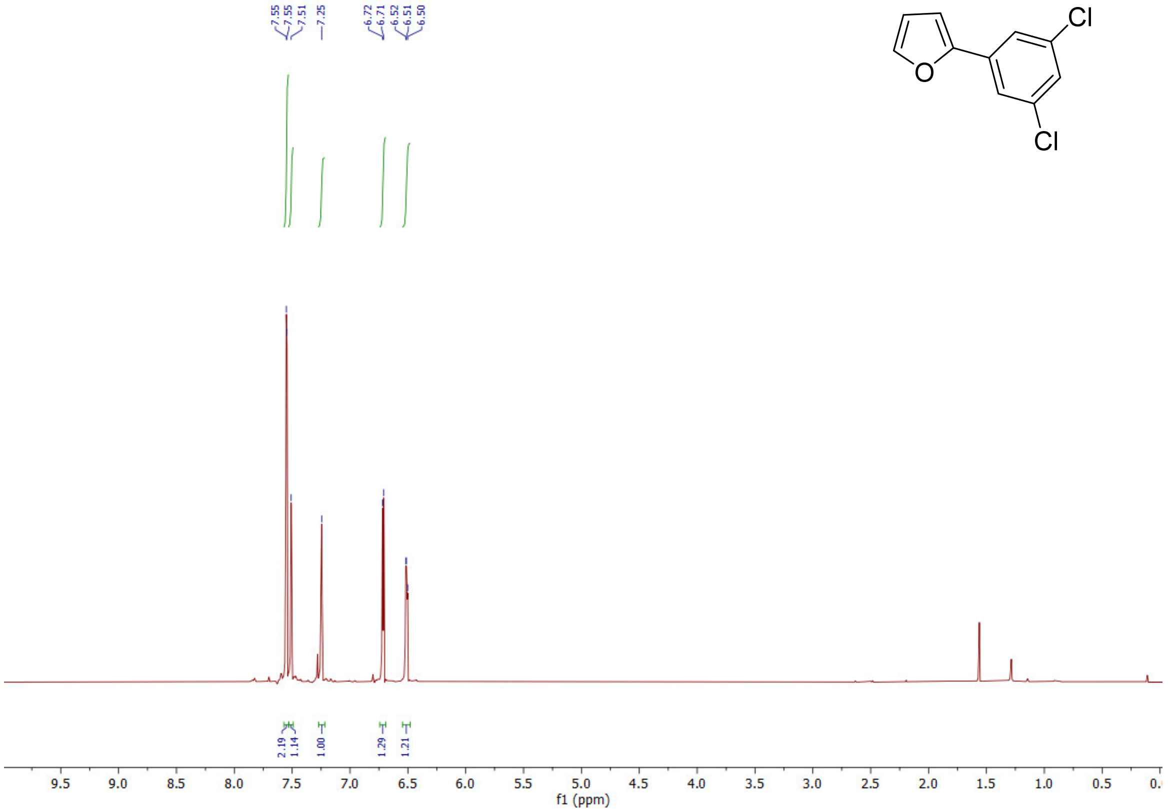

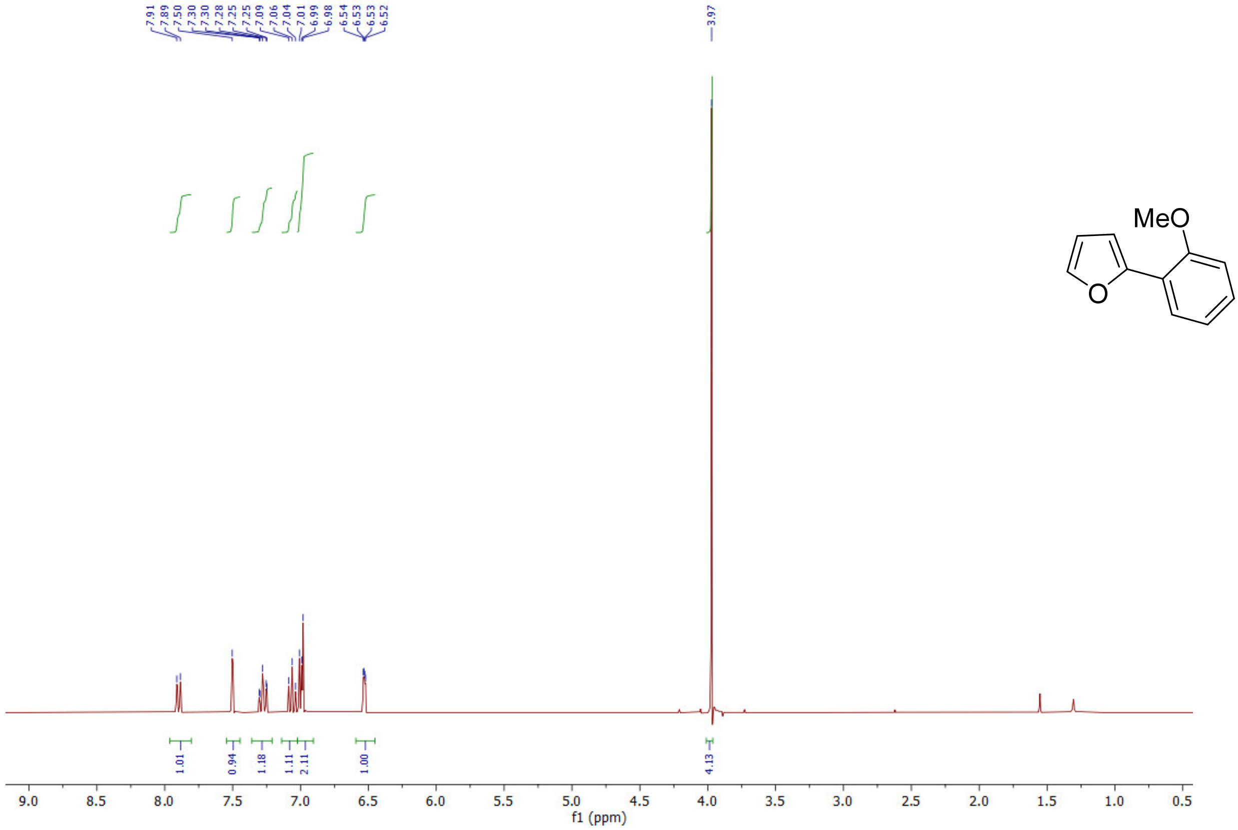

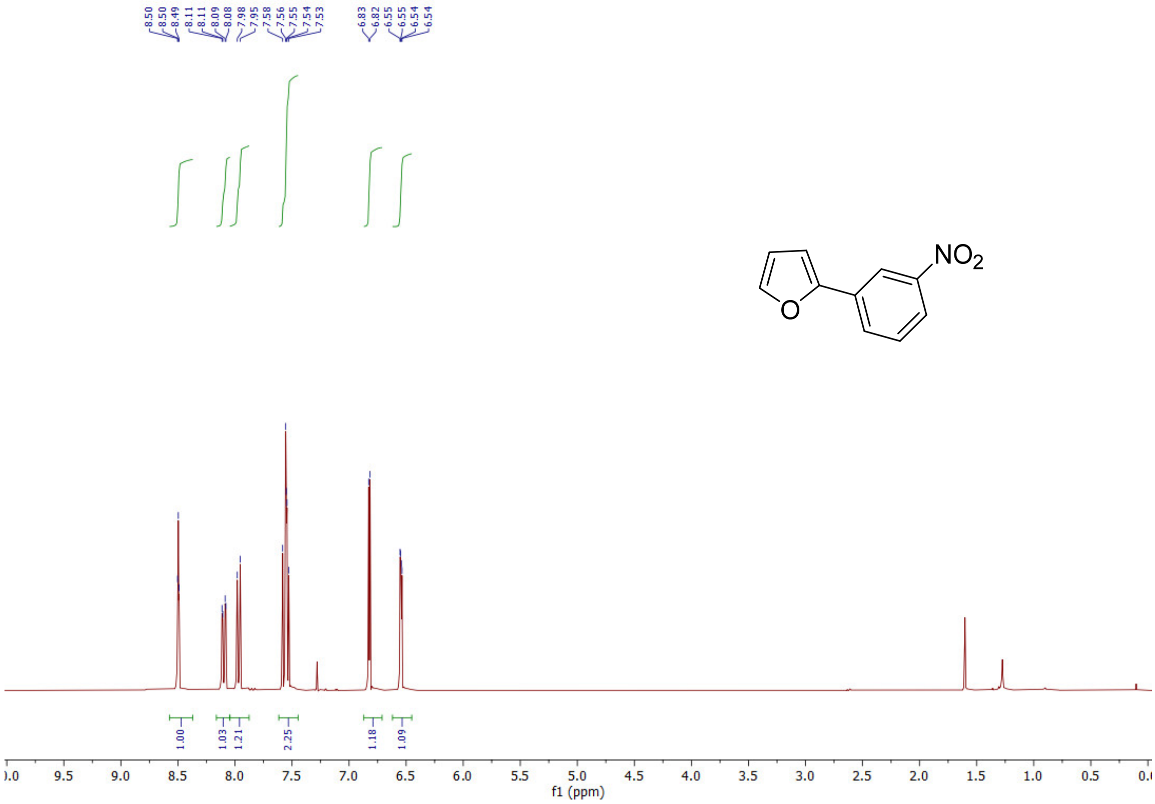
NO2
O
f1 (ppm)

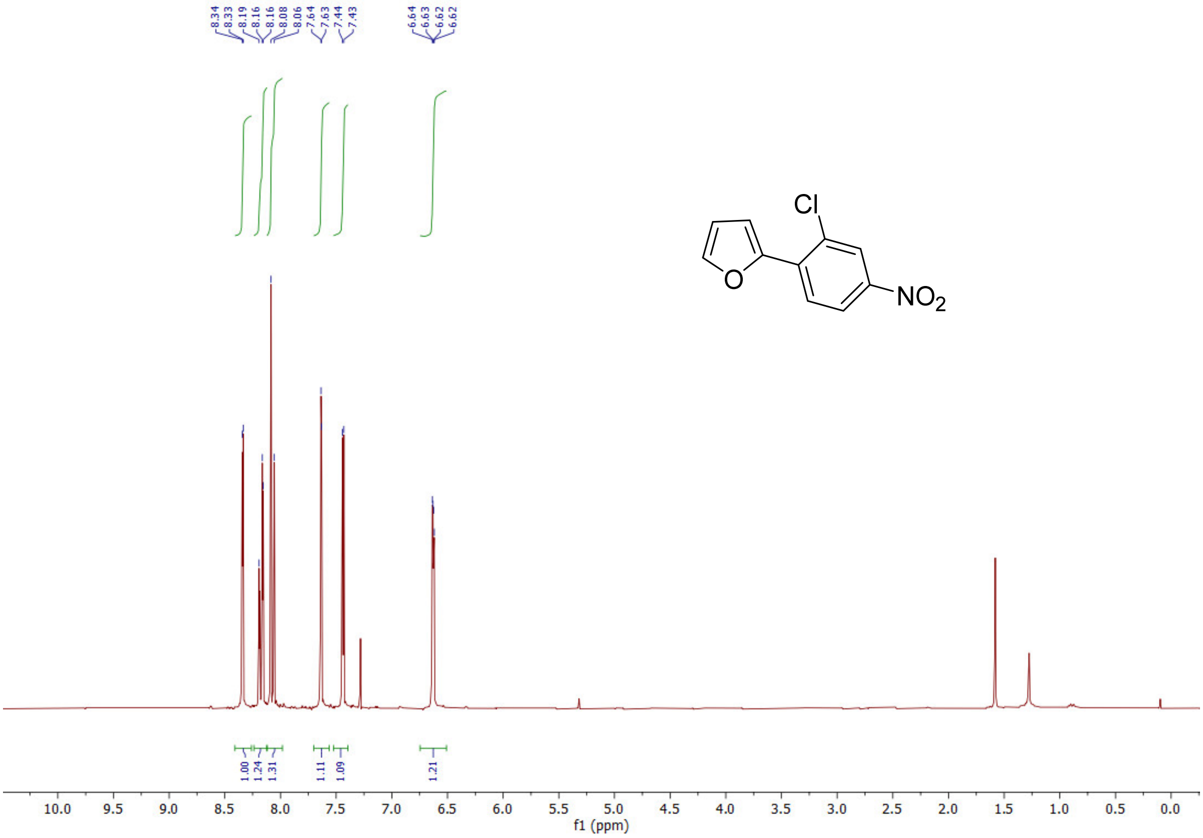
Cl
NO2
O
f1 (ppm)
